# Impact of Pre-Polymerization Thermal Modification on the Optical Resistance of Anterior Composites Against Thermal Cycling and Coffee Staining: An In Vitro Study

**DOI:** 10.3390/polym18161973

**Published:** 2026-08-13

**Authors:** Yasemin Gün, Hakan Yasin Gönder

**Affiliations:** Department of Restorative Dentistry, Faculty of Dentistry, Necmettin Erbakan University, 42090 Konya, Turkey

**Keywords:** color stability, preheated composite, supranofilled composite, resin-based composites, thermal aging

## Abstract

Color instability in anterior composite restorations remains a primary cause for clinical replacement. While pre-polymerization thermal modification is increasingly utilized in daily practice, its precise impact on phase-specific and long-term optical resistance against continuous thermal aging and aggressive dietary staining remains unclarified. This in vitro study evaluated the impact of pre-polymerization thermal modification (4 °C, 23 °C, and 55 °C) on the phase-specific and cumulative color stability (ΔE_00_) of four anterior composite resins subjected to sequential thermal aging and prolonged coffee immersion. One hundred twenty specimens (*n* = 10) were prepared, and ΔE_00_ was assessed using the CIEDE2000 formula at baseline (T0), post-thermal cycling (T1), and post-coffee immersion (T2). Mixed repeated-measures ANOVA revealed a significant three-way interaction (phase × material × temperature, *p* < 0.001), demonstrating that thermal conditioning effects vary by material formulation and aging dynamics. During thermal aging (ΔE_00_ T0–T1), pre-polymerization cooling (4 °C) induced significantly higher discoloration in Estelite Sigma Quick compared to preheated conditions (*p* < 0.001). Following coffee immersion (ΔE_00_ T1–T2), refrigeration at 4 °C significantly increased staining in Enamel Plus HRI (*p* = 0.026) and Estelite Sigma Quick (*p* = 0.007) compared to room temperature and preheated groups. For cumulative color change (ΔE_00_ T0–T2), the material type was the primary determinant (*p* < 0.001), while the independent effect of temperature was not statistically significant (*p* = 0.395). In conclusion, preheating (55 °C) provides no significant cumulative advantage against staining, whereas refrigeration (4 °C) increases susceptibility to physical and chemical discoloration. The intrinsic chemical composition remains the critical factor driving long-term color stability.

## 1. Introduction

Composite resin materials are extensively utilized in contemporary dental practice owing to their effective bonding to enamel and dentin, mechanical strength, cost-effectiveness, and ability to closely mimic natural tooth structure [1,2].

A fundamental requirement for any tooth-colored restoration is to accurately replicate the visual appearance of natural dentition, with clinical longevity relying primarily on initial shade matching followed by the material’s ongoing color permanence [3].

Despite continuous advancements, progressive discoloration resulting from prolonged exposure to the intraoral environment remains a major drawback [4]. In anterior restorations, such optical degradation directly compromises patient satisfaction and represents the primary cause of clinical failure and replacement [5]. Consequently, maintaining long-term color stability against physical and chemical oral challenges is critical for the clinical longevity of composite restorations [6].

Color alterations in composite resins are broadly classified into three distinct categories: extrinsic staining resulting from plaque buildup and superficial deposits; subsurface discoloration involving the light penetration and interaction of chromogenic agents within the outer composite layer (absorption); and intrinsic or bulk discoloration caused by internal physicochemical reactions occurring within the deeper matrix of the restoration [7].

Color alterations in resin-based composites can arise from intrinsic influences, extrinsic factors, or a combination of both [8]. Extrinsic discoloration occurs due to pigmentation on the restoration surface caused by plaque accumulation, surface deposits, consumption of staining food and beverages, and smoking [9,10]. A material’s susceptibility to intrinsic discoloration is governed by its constituent properties, notably the chemical structure of the resin matrix, filler loading, particle dimensions, and the extent of monomer conversion [11]. Furthermore, shifts in composite opacity over time contribute significantly to the long-term degradation of its optical aesthetics [12].

Despite advancements in the chemical composition and filler particles of composite resins, changes in color stability following polymerization have still been reported [13]. Consequently, various methods have been investigated to improve the physical and optical properties of resin-based restorative materials. One such method is the preheating of composite resins prior to polymerization [14].

Preheating has been reported to offer several advantages, including reduced material viscosity [15], increased monomer conversion rate [15,16], improved marginal adaptation [17,18], and shortened light-curing time [15]. Furthermore, this reduction in viscosity enhances the adaptation of the material to the cavity walls and minimizes marginal voids, potentially contributing to the long-term stability of the restoration by limiting the stresses associated with polymerization shrinkage [15]. The degree of conversion, defined as the conversion rate of carbon-carbon double bonds during polymerization, directly affects the mechanical properties, dimensional stability, solubility, biocompatibility, and color stability of composite resins [18]. Moreover, it has been reported that the temperature of the material prior to polymerization may significantly influence the degree of conversion [16].

Although numerous in vitro studies have examined how pre-polymerization thermal modification affects the physical and chemical properties of resin-based composites, the reported results remain inconsistent and inconclusive. While some authors have observed significant improvements in monomer conversion kinetics, surface microhardness, flexural strength, marginal integrity, and color stability, others have not reported similar improvements [19,20,21,22,23].

This gap in the literature is particularly notable concerning the interaction between various preheating temperatures and subsequent staining resistance under combined thermal aging and dietary challenges, which has not yet been sufficiently clarified. Therefore, evaluating color stability under these conditions was selected as the primary focus of this study, as aesthetic discoloration remains the principal clinical driver for the repair or replacement of anterior composite restorations.

This in vitro investigation aimed to assess the color stability of four contemporary anterior composite resins fabricated at three different pre-polymerization temperatures (4 °C, 23 °C, and 55 °C). In particular, the study focused on determining whether preheating before polymerization influences subsequent color alterations of composite resin materials after thermal aging and prolonged immersion in a coffee solution. In this manner, it was intended to determine the contribution of preheating to color stability, an esthetically critical parameter, and to provide scientific evidence to support clinical applications.

The null hypotheses tested in this study were as follows: The first null hypothesis was that the type of composite resin material would not significantly influence the color stability. The second null hypothesis was that different pre-polymerization temperatures (4 °C, 23 °C, and 55 °C) would not result in statistically significant differences in the color changes in the composite resins after thermal aging and exposure to a coffee solution.

## 2. Materials and Methods

### 2.1. Sample Size Calculation

The required sample size was initially determined using G*Power software (Version 3.1.9.6; Heinrich Heine University, Düsseldorf, Germany) for a factorial analysis of variance model including 12 experimental subgroups. Data from previous studies were analyzed to determine the appropriate sample size [3,24]. The calculation was based on an effect size (f) of 0.40, an alpha error probability of 0.05, and a target statistical power of 0.95. Accordingly, a total of 120 specimens, with 10 specimens in each experimental subgroup, were included in the study. The factorial design also allowed evaluation of the 6-df interaction terms relevant to the study aims, including the material × temperature interaction and the phase × material × temperature interaction.

### 2.2. Study Design

**Figure 1:**
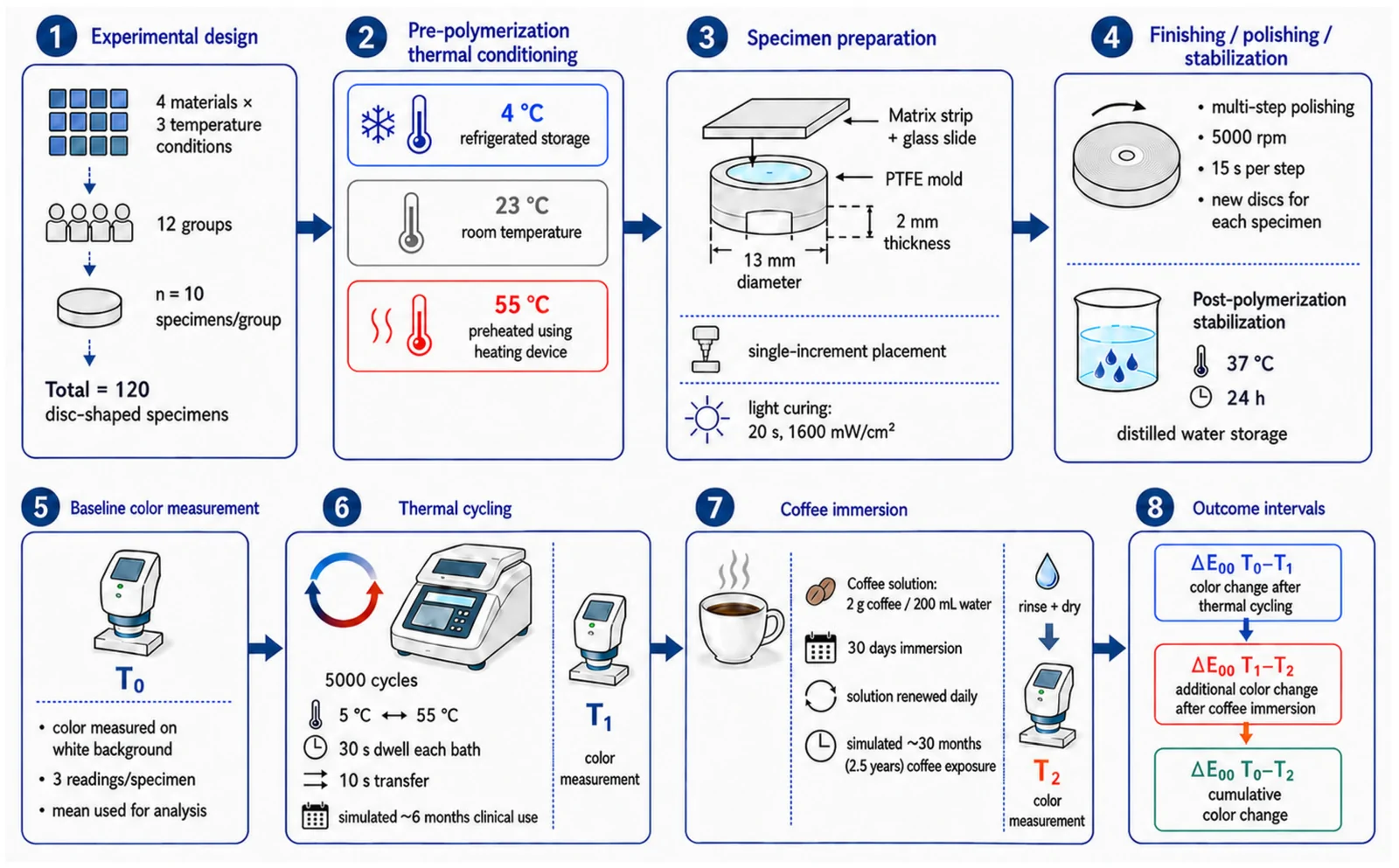
Study design (Created with ChatGPT 5.6).

The comprehensive experimental design, specimen preparation steps, thermal conditioning regimens, and outcome evaluation intervals are schematically illustrated in Figure 1. Figure 1, illustrating the study design flowchart, was generated with the assistance of ChatGPT (version 5.6).

This in vitro study was designed using a 4 × 3 factorial arrangement to evaluate the effects of four anterior composite resin materials and three pre-polymerization temperature conditions on color stability. The composite resin materials were Estelite Asteria^®^ (Tokuyama Dental, Tokyo, Japan), Estelite Sigma Quick^®^ (Tokuyama Dental, Tokyo, Japan), Filtek^™^ Ultimate (3M ESPE, St. Paul, MN, USA) and Enamel Plus HRI^®^ (Micerium, Genoa, Italy). The pre-polymerization temperature conditions were 4 °C, 23 °C, and 55 °C. These conditions were selected to represent refrigerated storage/cold placement, room temperature placement, and preheated placement, respectively.

A total of 120 disc-shaped specimens were prepared, with 10 specimens assigned to each of the 12 material-temperature combinations. Color measurements were performed at three time points: baseline (T0), after thermal cycling (T1), and after coffee immersion (T2). The primary phase-specific color change outcomes were defined as ΔE_00_ T0–T1, representing the color change after thermal aging, and ΔE_00_ T1–T2, representing the additional color change after coffee immersion. The ΔE_00_ T0–T2 interval was considered a secondary cumulative outcome reflecting the combined effect of thermal aging and coffee immersion.

The experimental groups were established as follows:

Group 1: Estelite Asteria^®^ (A2B, Tokuyama Dental, Tokyo, Japan) at 4 °C.

Group 2: Estelite Asteria^®^ (A2B, Tokuyama Dental, Tokyo, Japan) at 23 °C.

Group 3: Estelite Asteria^®^ (A2B, Tokuyama Dental, Tokyo, Japan) at 55 °C.

Group 4: Estelite Sigma Quick^®^ (A2, Tokuyama Dental, Tokyo, Japan) at 4 °C.

Group 5: Estelite Sigma Quick^®^ (A2, Tokuyama Dental, Tokyo, Japan) at 23 °C.

Group 6: Estelite Sigma Quick^®^ (A2, Tokuyama Dental, Tokyo, Japan) at 55 °C.

Group 7: Filtek^™^ Ultimate (A2B, 3M ESPE, St. Paul, MN, USA) at 4 °C.

Group 8: Filtek^™^ Ultimate (A2B, 3M ESPE, St. Paul, MN, USA) at 23 °C.

Group 9: Filtek^™^ Ultimate (A2B, 3M ESPE, St. Paul, MN, USA) at 55 °C.

Group 10: Enamel Plus HRI^®^ (UE2, Micerium, Genoa, Italy) at 4 °C.

Group 11: Enamel Plus HRI^®^ (UE2, Micerium, Genoa, Italy) at 23 °C.

Group 12: Enamel Plus HRI^®^ (UE2, Micerium, Genoa, Italy) at 55 °C.

### 2.3. Materials and Specimens’ Preparation

In this study, the A2 shades of Estelite Asteria^®^ (Tokuyama Dental, Tokyo, Japan), Estelite Sigma Quick^®^ (Tokuyama Dental, Tokyo, Japan), Filtek^™^ Ultimate (3M ESPE, St. Paul, MN, USA), and Enamel Plus HRI^®^ (Micerium, Genoa, Italy) composite resin materials, which are preferred for their aesthetic properties in anterior restorations, were utilized. A total of 120 disc-shaped specimens were prepared. The materials and devices used in the study are presented in Table 1 and Table 2.

Prior to polymerization, the specimens were conditioned at three different temperature values (4 °C, 23 °C, and 55 °C) based on clinical application and material stability standards. To optimize shelf life and maintain material stability, manufacturers typically advocate for cold storage within a temperature range of 4 °C to 20 °C; thus, the 4 °C group was designated to simulate these recommended preservation conditions, while the 23 °C group represented standard clinical ambient temperature [18,25,26,27]. Conversely, the selection of 55 °C as the preheating regimen was based on both thermodynamic efficiency and biological safety parameters. Increasing the polymerization temperature increases monomer conversion; however, this advantage operates within a defined thermal window, beyond which conversion rates decline—a critical threshold reported at approximately 90 °C for monomers such as Bis-GMA and Bis-EMA [28,29]. Although heating resins between 55 °C and 69 °C raises theoretical concerns regarding pulpal irritation, experimental evidence confirms that applying a composite preheated to 60 °C induces a negligible intrapulpal temperature rise of merely 0.8 °C, whereas standard 15-s light-curing exposure generates a substantially higher thermal increment of 4.5 °C to 5 °C [30]. Consequently, 55 °C was selected to ensure that pre-polymerization thermal modification remains well within clinically viable and biologically safe boundaries.

To achieve the determined temperatures, the composite resins assigned to the 4 °C group were stored in a refrigerator at 4 °C, while the room temperature group was kept in a temperature-controlled room at 23 °C for 2 h. For the 55 °C group, the materials were preheated to 55 °C using a Ronvig Ease-it^®^ (Rønvig Dental Mfg. Ltd., Daugaard, Denmark) composite resin heating device in accordance with the manufacturer’s instructions.

Once the target temperatures were reached, the composite resins were placed within 10 s into polytetrafluoroethylene (PTFE) molds with circular cavities measuring 13 mm in diameter and 2 mm in thickness using a single-increment technique [24].

To standardize the specimen surfaces and minimize the formation of an oxygen-inhibited layer, transparent matrix strips were placed on both sides of the mold, and a glass slide was gently pressed onto the upper surface to remove excess material [24]. Subsequently, light-curing was performed for 20 s at a light intensity of 1600 mW/cm^2^ using an LED (Light Emitting Diode) curing unit (Vega LED^®^, Dentac, İstanbul, Türkiye) operating in soft-start mode, in accordance with the manufacturer’s instructions.

Following polymerization, finishing and polishing procedures were performed on all specimens under dry conditions using a multi-step disc system (OptiDisc^®^, Kerr, Danbury, CT, USA) at a speed of 5000 rpm. Each polishing step lasted 15 s, new discs were used for each specimen, and all polishing steps were completed by the same operator to ensure consistency. Following the polishing process, the specimens were immersed in distilled water and stored at 37 °C for 24 h to allow post-polymerization reactions to stabilize.

### 2.4. Thermocycling Procedure

Subsequently, to simulate approximately six months of clinical use in the oral cavity, the specimens were subjected to an aging process of 5000 cycles in a thermal cycling device (the 1100^®^, SD Mechatronik Thermocycler, Feldkirchen-Westerham, Germany). The procedure involved dwelling in water baths at 5 °C and 55 °C for 30 s each, with a transfer time of 10 s between baths [31].

### 2.5. Coffee Immersion

Following the post-thermocycling color measurements, the specimens were immersed in a coffee solution (2 g Nescafe^®^ Classic (Nestlé S.A., Vevey, Switzerland)/200 mL water) prepared according to the manufacturer’s instructions for a period of 30 days to simulate 30 months (2.5 years) of clinical coffee consumption. To evaluate the long-term color permanence of the materials against severe extrinsic staining, this prolonged immersion protocol was established to simulate approximately 2.5 years (30 months) of functional clinical coffee consumption. Based on intraoral exposure models a continuous 24-h in vitro immersion corresponds to 1 month of in vivo exposure [32]. The coffee solution was renewed daily throughout the process, at the end of which the specimens were rinsed with distilled water and dried.

### 2.6. Color Measurements and ΔE_00_ Calculation

Color measurements were performed at three time points: baseline (T0), after thermal cycling (T1), and after coffee immersion (T2). Measurements were obtained using a digital colorimeter (PCE-CSM 6^®^, PCE Instruments, Meschede, Germany) on a standard white background under standardized conditions. Before each measurement session, the device was calibrated according to the manufacturer’s instructions. For each specimen and time point, L*, a*, and b* values were recorded three times, and the mean value was used for analysis [33].

Color differences were calculated using the CIEDE2000 formula [34]. Three ΔE_00_ intervals were calculated for each specimen: ΔE_00_ T0–T1, ΔE_00_ T1–T2, and ΔE_00_ T0–T2. The ΔE_00_ T0–T1 interval represented color change after thermal aging. The ΔE_00_ T1–T2 interval represented additional color change after coffee immersion. The ΔE_00_ T0–T2 interval was interpreted as a cumulative color change reflecting the combined effect of thermal aging and coffee immersion, rather than coffee staining alone.

### 2.7. Statistical Analysis

Statistical analyses were performed using JASP software (version 0.95.4; Amsterdam, the Netherlands). Descriptive data were reported as mean ± standard deviation. The primary analysis was performed using a mixed repeated-measures ANOVA. Experimental phase was defined as the within-subject factor with two levels: thermal aging color change (ΔE_00_ T0–T1) and coffee immersion-related additional color change (ΔE_00_ T1–T2). Composite resin material and pre-polymerization temperature conditions were included as between-subject factors. The model included the main effects of phase, material, and temperature, as well as all two-way and three-way interaction terms. Graphpad Prism (version 10.6.1 GraphPad Software, Boston, MA, USA) was used for data visualization.

Because a significant phase × material × temperature interaction was observed, simple main effects were evaluated to determine the effect of pre-polymerization temperature within each material and experimental phase, and the effect of material within each temperature condition and experimental phase. Homogeneity of variances was assessed using Levene’s test. Since Levene’s test indicated heterogeneity of variances, pairwise comparisons among material–temperature combinations were performed using Welch ANOVA and Games–Howell post-hoc tests.

The ΔE_00_ T0–T2 interval was analyzed separately as a secondary cumulative outcome using two-way ANOVA with composite resin material and pre-polymerization temperature condition as between-subject factors. The interaction between material and temperature was also included in the model. For all statistical analyses, the significance level was set at *p* < 0.05. Effect sizes were reported as partial eta squared (η_p_^2^).

## 3. Results

**Figure 2:**
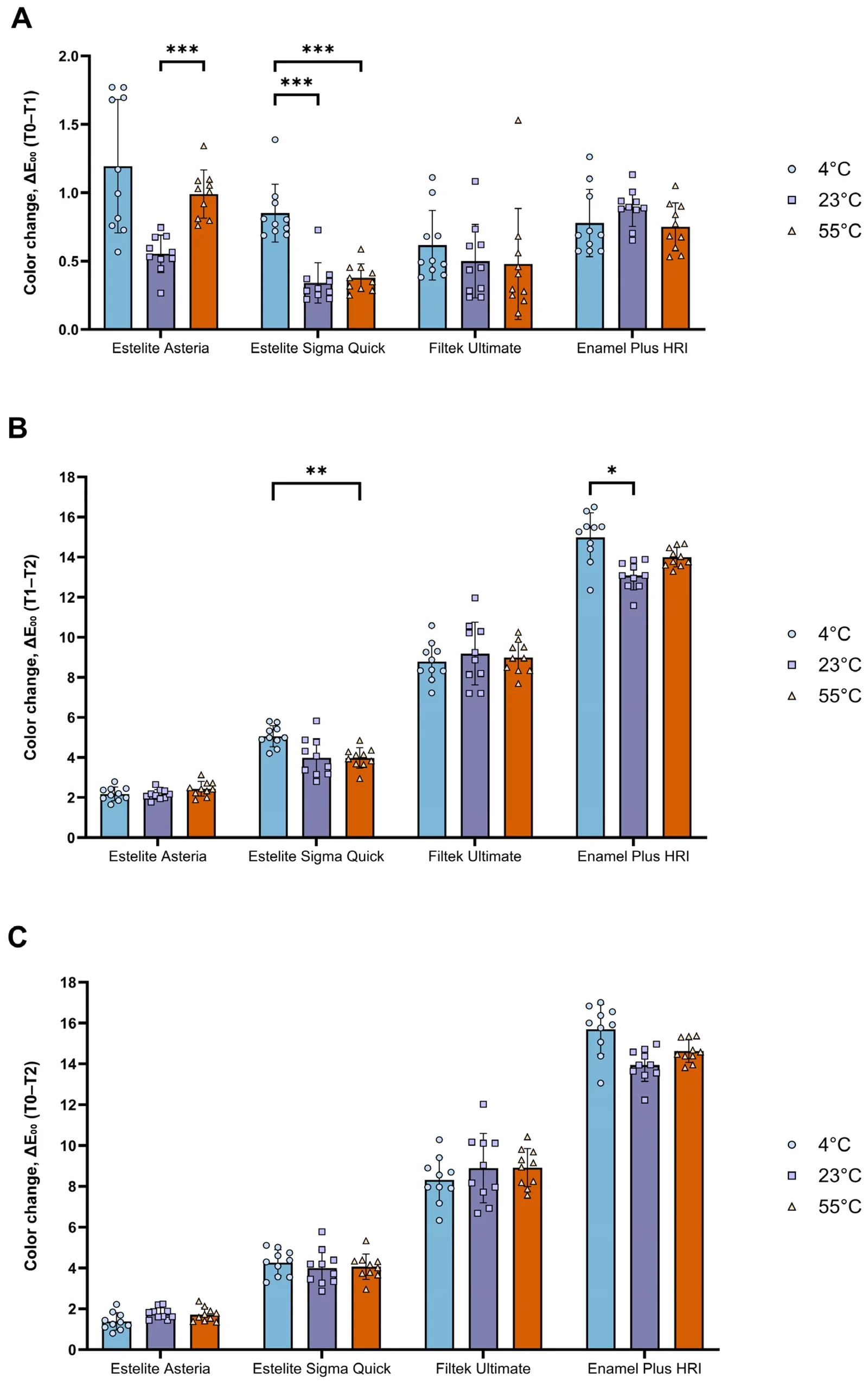
Phase-specific and cumulative color change values according to composite resin material and pre-polymerization temperature condition. (**A**) Color change after thermal aging, expressed as ΔE_00_ T0–T1. (**B**) Additional color change after coffee immersion, expressed as ΔE_00_ T1–T2. (**C**) Cumulative color change from baseline to after coffee immersion, expressed as ΔE_00_ T0–T2, reflecting the combined effect of thermal aging and coffee immersion. Data are presented as mean ± standard deviation with individual specimen values. Blue circles represent the 4 °C condition, purple squares represent the 23 °C condition, and orange triangles represent the 55 °C condition. Pairwise comparisons were performed using Games–Howell post-hoc tests. * *p* < 0.05, ** *p* < 0.01, *** *p* < 0.001.

In this study, color change values of four anterior composite resin materials conditioned at three different pre-polymerization temperatures were evaluated at two phase-specific intervals and one cumulative interval. The ΔE_00_ T0–T1 interval represented color change after thermal aging, whereas the ΔE_00_ T1–T2 interval represented additional color change after coffee immersion. The ΔE_00_ T0–T2 interval was evaluated separately as a secondary cumulative outcome. The phase-specific and cumulative color change values are presented in Figure 2.

### 3.1. Overall Phase-Specific Analysis

The primary phase-specific analysis was performed using a mixed repeated-measures ANOVA, with experimental phase as the within-subject factor and composite resin material and pre-polymerization temperature condition as between-subject factors. The results of the mixed repeated-measures ANOVA are presented in Table 3.

The analysis revealed a significant main effect of phase (F(1, 108) = 6809.267, *p* < 0.001, η_p_^2^ = 0.984), indicating that color change differed markedly between the thermal aging and coffee immersion phases. Significant phase × material (F(3, 108) = 1039.781, *p* < 0.001, η_p_^2^ = 0.967) and phase × material × temperature interactions (F(6, 108) = 4.938, *p* < 0.001, η_p_^2^ = 0.215) were also observed, indicating that the effect of pre-polymerization temperature depended on both composite resin material and experimental phase. The phase × temperature interaction was not statistically significant (F(2, 108) = 1.695, *p* = 0.188, η_p_^2^ = 0.030).

Among the between-subject effects, composite resin material (F(3, 108) = 1123.315, *p* < 0.001, η_p_^2^ = 0.969), pre-polymerization temperature condition (F(2, 108) = 12.392, *p* < 0.001, η_p_^2^ = 0.187), and the material × temperature interaction (F(6, 108) = 4.151, *p* < 0.001, η_p_^2^ = 0.187) were statistically significant. These findings indicate that phase-specific color change was primarily material-dependent, while the influence of pre-polymerization temperature varied according to the material and experimental phase.

### 3.2. Color Change After Thermal Aging: ΔE_00_ T0–T1

Color change values after thermal aging are shown in Figure 2A and summarized in Table 4. Overall, ΔE_00_ T0–T1 values remained relatively low across all material–temperature combinations. The highest mean ΔE_00_ T0–T1 value was observed in Estelite Asteria at 4 °C, whereas the lowest value was observed in Estelite Sigma Quick at 23 °C.

Simple main effect analysis showed that the effect of pre-polymerization temperature during the thermal aging phase was significant for Estelite Asteria (F = 11.184, *p* < 0.001) and Estelite Sigma Quick (F = 31.567, *p* < 0.001), but not for Filtek Ultimate (F = 0.169, *p* = 0.846) or Enamel Plus HRI (F = 1.497, *p* = 0.242). Games–Howell post-hoc comparisons showed that, in Estelite Asteria, the 23 °C group had significantly lower ΔE_00_ T0–T1 values than the 55 °C group (*p* < 0.001). In Estelite Sigma Quick, the 4 °C group showed significantly higher ΔE_00_ T0–T1 values than both the 23 °C and 55 °C groups (both *p* < 0.001). No significant temperature-related differences were detected within Filtek Ultimate or Enamel Plus HRI during the thermal aging phase.

### 3.3. Additional Color Change After Coffee Immersion: ΔE_00_ T1–T2

Additional color change after coffee immersion is shown in Figure 2B and summarized in Table 4. Compared with the thermal aging phase, ΔE_00_ T1–T2 values increased markedly in all groups. The lowest ΔE_00_ T1–T2 values were observed in Estelite Asteria across all temperature conditions, whereas Enamel Plus HRI showed the highest additional color change.

Simple main effect analysis showed that the effect of pre-polymerization temperature during the coffee immersion phase was significant for Enamel Plus HRI (F = 12.046, *p* < 0.001) and Estelite Sigma Quick (F = 8.113, *p* = 0.002), but not for Estelite Asteria (F = 2.326, *p* = 0.117) or Filtek Ultimate (F = 0.298, *p* = 0.744). Games–Howell post-hoc comparisons showed that, in Enamel Plus HRI, the 4 °C group had significantly higher ΔE_00_ T1–T2 values than the 23 °C group (*p* = 0.026). In Estelite Sigma Quick, the 4 °C group showed significantly higher ΔE_00_ T1–T2 values than the 55 °C group (*p* = 0.007). No significant temperature-related differences were detected within Estelite Asteria or Filtek Ultimate during the coffee immersion phase.

Material-related differences were significant at each pre-polymerization temperature condition during the coffee immersion phase: 23 °C (F = 252.390, *p* < 0.001), 4 °C (F = 428.185, *p* < 0.001), and 55 °C (F = 876.005, *p* < 0.001). At all three temperature conditions, Estelite Asteria showed the lowest additional color change, followed by Estelite Sigma Quick and Filtek Ultimate, while Enamel Plus HRI showed the highest additional color change.

### 3.4. Secondary Cumulative Color Change: ΔE_00_ T0–T2

Cumulative color change values from baseline to after coffee immersion are shown in Figure 2C and summarized in Table 4. This interval reflects the combined effect of thermal aging and coffee immersion. Two-way ANOVA revealed a significant main effect of composite resin material on cumulative ΔE_00_ T0–T2 values (F(3, 108) = 1282.074, *p* < 0.001, η_p_^2^ = 0.973). The main effect of pre-polymerization temperature condition was not statistically significant (F(2, 108) = 0.936, *p* = 0.395, η_p_^2^ = 0.017). However, the material × temperature interaction was significant (F(6, 108) = 3.798, *p* = 0.002, η_p_^2^ = 0.174).

Estelite Asteria exhibited lower cumulative discoloration values (ΔE_00_ =1.38 at 4 °C, 1.77 at 23 °C, and 1.72 at 55 °C) compared to all other composite groups across all temperatures (*p* < 0.05).

For cumulative ΔE_00_ T0–T2 values, Estelite Asteria showed the lowest color change across all temperature conditions, whereas Enamel Plus HRI showed the highest cumulative color change. Games–Howell post-hoc comparisons showed a borderline difference between the 4 °C and 23 °C conditions within Enamel Plus HRI (*p* = 0.050), whereas no significant temperature-related differences were observed within Estelite Asteria, Estelite Sigma Quick, or Filtek Ultimate.

## 4. Discussion

In this in vitro study, the impact of various pre-polymerization temperatures (4 °C, 23 °C, and 55 °C) on the color stability (ΔE_00_) of four contemporary anterior composite resins was evaluated across two distinct challenge phases: structural thermal aging (ΔE_00_ T0–T1) and long-term coffee immersion (ΔE_00_ T1–T2), alongside their cumulative interaction (ΔE_00_ T0–T2). According to the mixed repeated-measures ANOVA, a highly significant three-way interaction (phase × material × temperature, *p* < 0.001) was detected. This finding demonstrates that the effect of pre-polymerization temperature is not uniform; rather, it is dynamically modulated by both the specific material composition and the particular experimental phase. Consequently, both null hypotheses were explicitly rejected.

It was observed that the color changes following thermal aging remained within clinically acceptable limits for most materials; however, significant staining occurred in all composite resins after exposure to coffee solution. This finding underscores the potent staining potential of coffee and emphasizes the importance of utilizing such agents to simulate long-term dietary habits in restorative dentistry [9,10].

In the present study, a 30-day coffee immersion protocol with daily solution renewal was employed to simulate approximately 2.5 years of clinical coffee consumption, offering a standardized and rigorous chromogenic challenge [24,35].

The theoretical foundation of pre-heating relies on reducing the material’s viscosity to increase monomer kinetic energy, thereby facilitating a higher degree of polymerization conversion [36]. It is hypothesized that an increased degree of conversion may enhance chemical stability and potentially improve resistance to extrinsic staining [15,16,24].

The phase-specific findings revealed that these thermal mechanisms do not manifest as a generalized trend across all dental composites but are highly material-dependent. During the initial thermal aging phase (ΔE_00_ T0–T1), color changes remained relatively low across all combinations. However, simple main effect analysis indicated that temperature alterations significantly impacted the optical stability of Estelite Asteria (*p* < 0.001) and Estelite Sigma Quick (*p* < 0.001). Notably, for Estelite Sigma Quick, cooling the material to 4 °C resulted in significantly higher discoloration compared to both room temperature and preheated conditions (*p* < 0.001). In contrast, the subsequent coffee immersion phase (ΔE_00_ T1–T2) imposed a profound chromogenic stress, causing marked discoloration where all ΔE_00_ values exceeded the clinical acceptability threshold.

The isolated analysis of the coffee immersion phase (ΔE_00_ T1–T2) indicates that the temperature effect during this staining phase was highly significant for Enamel Plus HRI (*p* < 0.001) and Estelite Sigma Quick (*p* = 0.002). For Enamel Plus HRI, refrigeration at 4 °C significantly increased coffee discoloration compared to the 23 °C group (*p* = 0.026), while for Estelite Sigma Quick, the 4 °C group exhibited significantly higher staining than the 55 °C preheated group (*p* = 0.007). These outcomes indicate that while preheating offers localized benefits in reducing subsequent pigment absorption for specific formulations, pre-application cooling increases the material’s susceptibility to chemical staining.

Ultimately, when evaluating the cumulative color change (ΔE_00_ T0–T2), the two-way ANOVA confirmed a significant main effect of the composite resin material type (*p* < 0.001, η_p_^2^ = 0.973), whereas the independent main effect of temperature was not statistically significant (*p* = 0.395). Across all phases and temperature conditions, Estelite Asteria consistently demonstrated the lowest cumulative color change, whereas Enamel Plus HRI exhibited the highest cumulative values. This distinct separation highlights that specific material formulations exert a decisive control over esthetic survival. Across all thermal conditions, the submicron spherical filler system evaluated in this study consistently maintained superior surface smoothness, thereby minimizing pigment retention. In contrast, composite formulations with micro-hybrid architectures demonstrated a higher susceptibility to discoloration. These optical differences suggest that intrinsic matrix chemistry and filler geometry play a far more dominant role in long-term color stability than pre-polymerization thermal modifications alone. Furthermore, these material-specific variations are reinforced by differences in organic matrix formulations and filler loading, as composites with higher volumetric filler content (71 vol%) and simplified Bis-GMA/TEGDMA monomer systems exhibited superior optical permanence compared to those with lower filler volume fractions (63–63.3 vol%) incorporating multi-monomer resins such as UDMA or Bis-EMA.

The material-dependent nature of the findings aligns with Aktuğ Karademir et al. (2025) and Oskoee et al. (2022), who reported that the efficacy of thermal conditioning is contingent upon specific manufacturer formulations and matrix chemistry rather than exerting a universal effect [37,38]. However, the phase-specific analysis introduces a further distinction regarding the role of pre-polymerization cooling (4 °C).

The observation that preheating does not produce a uniform or cumulative improvement in optical properties across all groups is consistent with Mundim et al. (2011) and Çubukcu et al. (2024) [16,39]. These investigators reported that while pre-polymerization heating significantly enhances the depth of cure and monomer conversion kinetics due to reduced material viscosity, it does not necessarily translate into a direct, long-term reduction in extrinsic discoloration. Similarly, studies by Farghal et al. (2025) have emphasized that pre-heating has no significant effect on color stability, noting that color change is primarily dependent on the type of beverage and the hydrophilic nature of the resin matrix [40].

Furthermore, recent research conducted in 2026 continues to emphasize the material-dependent nature of preheating protocols. For instance, Takagi et al. (2026) demonstrated that pre-polymerization heating significantly reduces the viscosity of both flowable and conventional composite resins; however, the resulting mechanical performance and structural integrity vary considerably across different filler systems, suggesting that thermal conditioning cannot be assumed to yield uniform physical outcomes across all formulations [41].

The cumulative findings regarding the minimal overall impact of pre-polymerization temperature contrast with several reports in the literature that advocate for the benefits of preheating protocols. Specifically, Darabi et al. (2019), Gonulol & Karaman (2013), Sousa et al. (2015), and Matani et al. (2024) demonstrated that preheating resin composites prior to light activation significantly enhances their subsequent color stability when exposed to chromogenic media such as tea and coffee, these researchers attributed the improved stain resistance to an elevated degree of monomer conversion and a more highly cross-linked polymer network, which theoretically limits the penetration of extrinsic pigments [24,42,43,44]. However, the lack of a statistically significant cumulative temperature main effect (ΔE_00_ T0–T2, *p* = 0.395) observed in the present study indicates that such benefits may not be universal across all contemporary material formulations.

The lack of a cumulative clinical advantage from preheating protocols is further supported by the structural and chemical mechanisms described by Daneshpooy et al. (2024) and Kahnamouei et al. (2017), these researchers reported that, although preheating prior to polymerization initially reduces material viscosity and thus facilitates processing, the thermal stress caused by the preheating process could potentially alter the internal network structure of certain composite formulations, which might partially offset the initial benefits achieved through enhanced monomer mobility [45,46].

In this study, the CIEDE2000 color difference formula, currently accepted as the gold standard in the contemporary literature for most accurately reflecting human color perception, and clinical threshold values (PT = 0.8, AT = 1.8) were utilized to concretize the clinical relevance of our data [47].

This observation suggests that while thermal conditioning may alter initial resin kinetics, overall color preservation is more complexly linked to the inherent structural properties and surface dynamics of individual material formulations. Clinicians restoring aesthetic anterior zones should prioritize composite formulations demonstrating high intrinsic stain resistance, particularly for patients with frequent coffee consumption habits. While preheating offers clinical handling and adaptation benefits during placement, clinicians should not rely on pre-conditioning thermal protocols alone to guarantee superior long-term optical stability if a material inherently susceptible to discoloration is selected.

Certain limitations of this investigation should be taken into account when evaluating the findings. First, as an in vitro experimental study, the complex and dynamic conditions of the oral environment could not be fully replicated. Factors such as salivary enzymes, protective pellicle formation, biofilm accumulation, mechanical wear, and pH fluctuations were not included, all of which significantly influence the color stability of composite resins in vivo [43,48].

Second, the 30-day continuous coffee immersion protocol represents an aggressive staining model that differs from clinical reality. In a clinical zone, the intermittent nature of exposure to staining agents, combined with the mitigating effects of salivary clearance and routine oral hygiene practices, generally reduces the intensity of extrinsic staining [43,48]. By excluding these mitigating biological elements, the current split-phase protocol may have amplified the material-dependent differences in discoloration and potentially overshadowed more subtle, long-term influences of pre-polymerization temperatures. However, this standardized “worst-case scenario” was intentionally selected to assess the maximum staining potential and the inherent chemical stability of the composite resin matrix under accelerated and controlled conditions.

Third, only one chromogenic solution (coffee) was utilized to simulate extrinsic discoloration. Although coffee represents a commonly consumed beverage with high staining potential, the optical consequences of other dietary agents such as tea, red wine, or carbonated acidic drinks were not evaluated [24]. Fourth, a single preheating device with fixed temperature boundaries was investigated; variations in preheating duration, repeated heating cycles, or alternative thermal conditioning units may result in different outcomes.

Fifth, although the CIEDE2000 formula stands as a perceptually uniform method, parallel visual assessments by human observers were not conducted. Therefore, instrument-based measurements may present discrepancies when correlated with direct subjective clinical perception [47]. Sixth, due to the rapid heat loss that occurs when transferring the composite resin from the heating oven to the matrix mold, the exact temperature within the composite at the time of placement could not be dynamically monitored; this highlights a limitation inherent in bench-top preheating simulations [49].

Additionally, color stability was investigated as the primary outcome in this study, as optical degradation represents a main driver for patient dissatisfaction and clinical restoration replacement in anterior teeth [24]. However, a key limitation of this investigation is the absence of direct physical–chemical, mechanical, and structural characterizations—such as degree of conversion (DC) via Fourier Transform Infrared Spectroscopy (FTIR), water sorption, solubility, microhardness, flexural strength, cross-sectional morphological evaluations, and surface roughness parameters. While the observed optical trends are interpreted in light of known polymer network dynamics, these mechanistic explanations remain inferential without direct microstructural and morphological data. Given that monomer conversion, water uptake, and surface micro-topography directly govern pigment adsorption and matrix degradation, the clinical conclusions regarding pre-polymerization conditioning should be interpreted cautiously. Future multi-factorial studies evaluating both optical and physicochemical parameters simultaneously are warranted to definitively clarify the underlying mechanisms. Another limitation of the study is that, although the selected composite resins represent commonly used commercial categories, the evaluated formulations naturally limit the direct generalizability of the results to all resin systems.

Future lines of research should explore the long-term optical and mechanical durability of diverse preheated composite systems across standardized temperature ranges under both simulated physiological conditions and in vivo environments.

## 5. Conclusions

Based on the multi-phase statistical framework and the limitations of this in vitro investigation, it can be concluded that the optical behavior of direct aesthetic restorations is modulated by a significant three-way interaction between the specific material composition, the type of aging challenge, and the pre-polymerization temperature (*p* < 0.001). Pre-polymerization heating to 55 °C does not appear to provide a generalized or statistically significant cumulative advantage for preventing extrinsic discoloration, as its independent main effect on overall optical changes was not significant (*p* = 0.395).

Ultimately, when evaluating the overall aesthetic survival from baseline to the end of the prolonged coffee immersion (ΔE_00_ T0–T2), the findings suggest that the intrinsic chemical formulation and filler technology specified by the manufacturer act as the primary determinants driving long-term color stability (*p* < 0.001), while the independent role of clinical thermal pre-conditioning remains limited.

## Figures and Tables

**Table 1:** The materials used in this study.

Category	Material	Manufacturer	Type	Content	Filler Load (wt%/vol%)	Lot Number
Composite Resin	Estelite Asteria^®^	Tokuyama Dental, Tokyo, Japan	Supra-nanofilled	Bis-GMA, TEGDMA/Spherical silica-zirconia filler	82/71	290E15
Composite Resin	Estelite Sigma Quick^®^	Tokuyama Dental, Tokyo, Japan	Supra-nanofilled	Bis-GMA, TEGDMA/Spherical silica-zirconia filler	82/71	303 × 10^44^
Composite Resin	Filtek^™^ Ultimate	3M ESPE, St. Paul, MN, USA	Nanofilled	Bis-GMA, UDMA, TEGDMA, Bis-EMA/Non-agglomerated silica, cluster zirconia/silica	78.5/63.3	11,263,793
Composite Resin	Enamel Plus HRI^®^	Micerium S.p.A., Genoa, Italy	Micro-hybrid	Bis-GMA, UDMA/Zirconium dioxide glass filler	80/63	2,023,001,079
Polishing Discs	OptiDisc^®^	Kerr, Danbury, CT, USA	-	-	-	-

UDMA: urethane dimethacrylate; Bis-EMA: ethoxylated bisphenol-A dimethacrylate; TEGDMA: triethylene glycol dimethacrylate; Bis-GMA: bisphenol-A-glycidyl dimethacrylate.

**Table 2:** The devices used in the study.

Device Type	Model	Manufacturer
Composite Resin Heating Device	Ease-it^®^	Ronvig, Daugaard, Denmark
Curing Unit	Vega LED^®^	Dentac, Istanbul, Türkiye
Thermocycler	The 1100^®^	SD Mechatronik, Feldkirchen, Germany
Colorimeter	CSM 6^®^	PCE Instruments, Meschede, Germany

**Table 3:** Mixed repeated-measures ANOVA results for phase-specific ΔE_00_ values.

Effect	df	F	*p*	η_p_^2^
Within-subject effects				
Phase	1, 108	6809.267	<0.001	0.984
Phase × Material	3, 108	1039.781	<0.001	0.967
Phase × Temperature	2, 108	1.695	0.188	0.030
Phase × Material × Temperature	6, 108	4.938	<0.001	0.215
Between-subject effects				
Material	3, 108	1123.315	<0.001	0.969
Temperature	2, 108	12.392	<0.001	0.187
Material × Temperature	6, 108	4.151	<0.001	0.187

Phase included two levels: ΔE_00_ T0–T1, representing color change after thermal aging, and ΔE_00_ T1–T2, representing additional color change after coffee immersion. η_p_^2^: partial eta squared.

**Table 4:** Descriptive ΔE_00_ values and pairwise comparisons according to composite resin material and pre-polymerization temperature condition.

Outcome/Material	4 °C	23 °C	55 °C
**ΔE_00_ T0–T1/Thermal aging**			
Estelite Asteria	1.195 ± 0.488 ^ab,A^	0.554 ± 0.136 ^b,A^	0.990 ± 0.176 ^a,B^
Estelite Sigma Quick	0.851 ± 0.211 ^a,A^	0.341 ± 0.149 ^b,A^	0.379 ± 0.101 ^b,A^
Filtek Ultimate	0.617 ± 0.255 ^a,A^	0.500 ± 0.269 ^a,A^	0.579 ± 0.706 ^a,AB^
Enamel Plus HRI	0.779 ± 0.246 ^a,A^	0.891 ± 0.137 ^a,B^	0.751 ± 0.175 ^a,B^
**Δ** **E_00_ T1–T2/Coffee immersion**			
Estelite Asteria	2.185 ± 0.338 ^a,A^	2.160 ± 0.245 ^a,A^	2.439 ± 0.365 ^a,A^
Estelite Sigma Quick	5.068 ± 0.524 ^a,B^	3.990 ± 0.954 ^ab,B^	3.982 ± 0.509 ^b,B^
Filtek Ultimate	8.789 ± 0.967 ^a,C^	9.188 ± 1.562 ^a,C^	8.992 ± 0.795 ^a,C^
Enamel Plus HRI	14.984 ± 1.236 ^a,D^	13.077 ± 0.714 ^b,D^	13.998 ± 0.478 ^ab,D^
**ΔE_00_ T0–T2/Cumulative color change**			
Estelite Asteria	1.385 ± 0.430 ^a,A^	1.772 ± 0.281 ^a,A^	1.719 ± 0.338 ^a,A^
Estelite Sigma Quick	4.267 ± 0.632 ^a,B^	4.007 ± 0.870 ^a,B^	4.070 ± 0.619 ^a,B^
Filtek Ultimate	8.319 ± 1.118 ^a,C^	8.893 ± 1.697 ^a,C^	8.919 ± 0.929 ^a,C^
Enamel Plus HRI	15.699 ± 1.225 ^a,D^	13.942 ± 0.795 ^b,D^	14.630 ± 0.557 ^ab,D^

Data are presented as mean ± standard deviation. Lowercase superscript letters indicate pairwise comparisons among temperature conditions within the same material and outcome. Uppercase superscript letters indicate pairwise comparisons among materials within the same temperature condition and outcome. Values sharing the same superscript letter are not significantly different. Pairwise comparisons were performed using Games–Howell post-hoc tests. ΔE_00_ T0–T1 represents color change after thermal aging; ΔE_00_ T1–T2 represents additional color change after coffee immersion; ΔE_00_ T0–T2 represents cumulative color change reflecting the combined effect of thermal aging and coffee immersion.

## Data Availability

The original contributions presented in this study are included in the article. Further inquiries can be directed to the corresponding author.

## Abbreviations

The following abbreviations are used in this manuscript:

ANOVA	Analysis of Variance
AT	Acceptability Threshold
Bis-EMA	Ethoxylated Bisphenol-A Dimethacrylate
Bis-GMA	Bisphenol-A-Glycidyl Dimethacrylate
CIE	Commission Internationale de l’Éclairage
CIEDE2000	Commission Internationale de l’Éclairage 2000 Color Difference Formula
df	Degrees of Freedom
FTIR	Fourier Transform Infrared Spectroscopy
LED	Light-Emitting Diode
PT	Perceptibility Threshold
PTFE	Polytetrafluoroethylene
TEGDMA	Triethylene Glycol Dimethacrylate
UDMA	Urethane Dimethacrylate
η_p_^2^	Partial Eta Squared
ΔE_00_	CIEDE2000 Color Change Value
